# What Matters When It Comes to Trust in One's Physician: Race/Ethnicity, Sociodemographic Factors, and/or Access to and Experiences with Health Care?

**DOI:** 10.1089/heq.2019.0101

**Published:** 2020-06-29

**Authors:** Anthony L. Nguyen, Rebecca J. Schwei, Ying-Qi Zhao, Paul J. Rathouz, Elizabeth A. Jacobs

**Affiliations:** ^1^ Division of Hematology and Medical Oncology, Loma Linda University Health, Loma Linda, California, USA.; ^2^ BerbeeWalsh Department of Emergency Medicine, University of Wisconsin Madison School of Medicine and Public Health, Madison, Wisconsin, USA.; ^3^ Department of Biostatistics and Medical Informatics, University of Wisconsin Madison School of Medicine and Public Health, Madison, Wisconsin, USA.; ^4^ Department of Population Health, The University of Texas at Austin Dell Medical School, Austin, Texas, USA.; ^5^ Department of Medicine, The University of Texas at Austin Dell Medical School, Austin, Texas, USA.

**Keywords:** interpersonal trust, race/ethnicity, disparities, access, health care experiences

## Abstract

**Purpose:** Interpersonal trust is linked to therapeutic factors of patient care, including adherence to treatment, continuity with a provider, perceived effectiveness of care, and clinical outcomes. Differences in interpersonal trust across groups may contribute to health disparities. We explored whether differences in interpersonal trust varied across three racial/ethnic groups. Additionally, we explored how different health care factors were associated with differences in trust.

**Methods:** We conducted a cross-sectional, computer-administered survey with 600 racially and ethnically diverse adults in Chicago, IL, from a wide variety of neighborhoods. We used staged ordinal logistic regression models to analyze the association between interpersonal trust and variables of interest.

**Results:** Interpersonal trust did not differ by racial or ethnic group. However, individuals with 0–2 annual doctor visits, those reporting having a “hard time” getting health care services, those answering “yes” to “Did you not follow advice or treatment plan because it cost too much?,” and those reporting waiting more than 6 days/never getting an appointment had significantly increased odds of low trust. We did not find differences across racial/ethnic groups.

**Conclusion:** Our study suggests that access to health care and interactions within the health care setting negatively impact individual's trust in their physician.

## Background

Interpersonal trust in one's physician is a multidimensional concept^1^ that gives meaning and substance to the patient–doctor relationship. It differs from patient satisfaction because it is forward looking and reflects expectations of an ongoing relationship.^2,3^ While the definition of interpersonal trust varies, many agree that interpersonal trust is “accepted vulnerability to another's possible but not expected ill will.”^4^ Interpersonal trust is linked to many therapeutic factors of patient care, including adherence to treatment, continuity with a provider, perceived effectiveness of care, and clinical outcomes.^1,5–8^ A meta-analysis demonstrated a significant association between interpersonal trust and health outcomes such as health-related quality of life or patient satisfaction.^9^

Racial and ethnic disparities in interpersonal trust may contribute to health disparities across populations. In several studies, African American patients have been shown to have lower trust in physicians than white patients.^10–14^ A study by LaVeist et al. found that while both African American and white cardiac patients did not trust the health care system African American patients were significantly more likely to report mistrust in all measures compared with white patients.^13^ Likewise, in a population-based study, non-Hispanic black respondents were less likely than non-Hispanic white respondents to trust their physician.^10^ Similarly, Hispanics reported lower measures of indirect trust compared with non-Hispanic white populations^15^ and higher distrust than non-Hispanic whites.^16^

Other factors, including education, health literacy income, health status, physicians' communication style, and interpersonal skills have also been shown to be related to differences in interpersonal trust.^4,17,18^ One study reported that education, income, and health status influenced interpersonal trust in a physician; however, lack of continuity of care was more important in low levels of trust.^11^ Similarly, a review by Hall et al. suggests that the strongest predictors of interpersonal trust are physician communication style and interpersonal skills.^4^ These studies suggest that contextual factors, including the place where people usually get their care, continuity of care with a regular provider, and the quality of the interaction are the factors that are most related to interpersonal trust.^9,19,20^

We further explored this hypothesis by investigating how interpersonal trust was associated with various sociodemographic factors, including race/ethnicity. We also sought to understand how this relationship changed when we considered health care access factors and previous health care experiences. We hypothesized that both non-Hispanic black and Mexican Hispanic patients would have lower levels of interpersonal trust as compared with white patients, and that respondents who had difficulty accessing health care would also have lower trust.

## Methods

### Participants and data collection

We conducted a cross-sectional, computer-administered survey among a convenience sample of adults shopping at selected supermarkets in seven socioeconomically diverse neighborhoods in Chicago, IL. We recruited individuals who self-identified as non-Hispanic black, Mexican Hispanic, and non-Hispanic white with a range of sociodemographic characteristics. We did this by recruiting from grocery stores in neighborhoods of varying wealth. When we reached a sample of ∼200 participants from a particular racial/ethnic group, we no longer took volunteers from that group allowing us to achieve a target study sample close to 600 adults with equal proportions self-identifying as non-Hispanic black, Mexican Hispanic, and non-Hispanic white.

There were 25 participants who either left the race question blank (*n*=23) or self-identified as Asian (*n*=2). Because we were looking to compare interpersonal trust between non-Hispanic blacks, Mexican Hispanics, and non-Hispanic whites, these participants were dropped from our analysis.

All participants were asked at recruitment if they identified as non-Hispanic black, Mexican Hispanic, or non-Hispanic white. Participants were also asked if they self-identified as Spanish/Hispanic/Latino and, if yes, they were asked to select if they identified as Mexican, Mexican American, Chicano; Puerto Rican; Cuban; or other. Next all participants irrespective of ethnicity were asked to select a race—white; black, African American or Negro; Asian; or Other. For the purposes of this study if a participant marked that they self-identified as Mexican, Mexican American, or Chicano they were included in the Mexican Hispanic group irrespective of race. Participants were classified as non-Hispanic white if they marked that they were not of Spanish/Hispanic/Latino and marked that their race was white. Likewise participants were classified as non-Hispanic black if they marked that they were not of Spanish/Hispanic/Latino and marked that their race was black or African American.

Volunteers were eligible to participate if they were fluent in either English or Spanish, at least 18 years of age, and were able to give informed consent. The survey was administered in English or Spanish according to the participant's preference. The Cook County Institutional Review Board approved all study activities.

### Measures

The survey included questions regarding sociodemographic characteristics, health care access and usage, and interpersonal trust among other things. Interpersonal trust was measured using the validated Hall Trust Scale.^21^ The Hall Trust Scale is a 10-item Likert scale questionnaire that includes questions like: “Your doctor will do whatever it takes to get you all the care you need.” Answer options included strongly disagree, disagree, neutral (neither agree nor disagree), agree, and strongly agree.

We measured sociodemographic variables that have been shown to influence interpersonal trust.^4,17^ These included age, gender (male or female), marital status (married, previously married, single), employment status (employed, unemployed, or homemaker/retired/student), income (≤$15,999, $16,000–$34,999, $35,000–$74,999, or ≥$75,000), and education level (less than high school, high school/GED, trade school/associate's degree, or bachelor's degree and above).

Additionally, we measured insurance status, the participant's usual place of health care, and the number of physician visits in the past 12 months. The participant's insurance status was categorized as private/military insurance, Medicare/Medicaid, or no insurance. The participant's usual source of health care was categorized as a doctor's office, hospital/health system clinic, community clinic, or urgent care/emergency. The number of annual physician visits was categorized into 0–2, 3–5, and >6 annual doctor visits.

We also asked about participants' previous experiences in health care in the past 2 years, including: “In the last 2 years, how hard was it for you to get the health services needed?” Response options included “Hard,” “Not very hard,” and “Have not needed healthcare in the last 2 years.” To understand whether or not respondents avoided seeking health care due to cost we asked participants “Was there any time in the last 2 years when you did not seek medical care because it was too expensive or health insurance did not cover it?” Response options included “No,” “Yes,” or “Not sure.” 

We also asked how long they had to wait to be seen by a physician by asking them “Now think about all your health care experiences over the last two years. The last time you were sick or needed medical attention, how quickly could you get an appointment to see a doctor or health professional?” Response options included: “Same Day,” “Next Day,” “2–3 days,” “4–5 days,” “6–7 days,” “ > 1week,” “never able to get appointment,” and “did not seek care in the past 2 years or went to ER/Urgent Care/No Apt.” These responses were collapsed into three categories: <6 days, >6 days, or “did not seek care in the last 2 years.”

Participants were asked “In the last 2 years, has there been a time when you did not follow advice or treatment plan because of cost? Response options include “Yes,” “No,” and “Do not know.”

### Analysis

Because this analysis was focused on interpersonal trust, the analysis only included respondents who identified someone as their personal doctor and had seen them in the last year. To calculate an overall interpersonal trust score, each text response was given a value between 1 and 5. A score of 1 indicated high trust and a score of 5 indicated low trust. We summed the values of the 10 items in the scale. When indicated, items were reverse coded so a response of “never true” indicated lower trust and received a score of 5.

The objective of our study was to assess what factors were associated with low interpersonal trust, and therefore higher numbers indicated lower trust to facilitate interpretation. The possible range of score values was 10–50, with higher values indicating lower interpersonal trust. Thirty respondents did not answer 1 of the 10 questions, six did not answer 2 questions, and three did not answer 4 questions. We imputed missing data using multiple imputation using SAS PROC MI, which is based on a multivariate normal approach by drawing all the variables from a multivariate normal distribution in the imputation model.^22^ The imputation model included those variables to be used in the analysis model, and thus all relevant variables were considered.

The trust scores were distributed into tertiles with natural peaks in the distribution of scores at scores of 10, 20, and 30. Therefore, we created a trust variable with three levels of trust: high (10–20), medium (21–29), and low (30–50) trust. Making the trust score a categorical variable also facilitated interpretation.

We compared sociodemographic variables, health care access variables, and previous experiences with health care using chi-square tests for categorical variables and one-way analysis of variance (ANOVA) for continuous variables across racial and ethnic groups. We also examined whether there were differences in the categorical interpersonal trust variable across the three racial and ethnic groups using a chi-square test.

Next, we used a staged ordinal logistic regression using STATA 13 to determine how the relationship between race/ethnicity and interpersonal trust was modified by the other variables of interest. We created three models. The variables included in each of the three models are listed in Table 1. In addition to running Model 3 for the entire sample, we also ran Model 3 for each racial/ethnic group. Finally, to ensure that our outcomes were the same when we collapsed the annual number of physician visits and time to getting an appointment into fewer categories, we conducted a sensitivity analysis.

**Table 1.** Description of the Variables Included in the Various Ordinal Logistic Regression Models

	Model 1	Model 2	Model 3
**Sociodemographic variables:** Race/ethnicity, age, sex, marital status, employment status, family income, education level	X	X	X
**Health care access variables:** Insurance status, no. of annual visits; health care setting		X	X
**Previous experiences with health care variables:** Challenges getting health services, did not seek care due to cost, time to appointment, did not follow advice because it cost too much			X

## Results

All 598 respondents are included in Table 2, which describes the characteristics of the population. More than 34% of respondents self-identified as non-Hispanic black, 32% as non-Hispanic white, and 32% as Mexican Hispanic. The average ages of respondents in the three racial/ethnic groups were 41.8, 36.4, and 42.7 years, respectively. Across all racial/ethnic groups, close to 60% of our respondents self-identified as female. Mexican Hispanic respondents were more likely to be married as compared with non-Hispanic black and non-Hispanic white participants. Non-Hispanic blacks were more likely to be unemployed and have an annual family income of less than $15,999 compared with Mexican Hispanics and non-Hispanic whites. Non-Hispanic whites were more likely to have a degree above high school as compared with non-Hispanic blacks and Mexican Hispanics.

**Table 2.** Sociodemographic, Health Care Access, and Health Care Use Characteristics of Entire Sample Across Three Racial Ethnic Groups, % (*n*)

Study variables	Overall (*n*=598)	Non-Hispanic Black (*n*=208)	Mexican Hispanic (*n*=194)	Non-Hispanic White (*n*=196)	*p*^a^
Mean age (SD), years	40.3 (14.2)	41.8 (11.9)	36.4(13.8)	42.7 (15.7)	<0.001^b^
Female	58.6 (346)	57.9 (117)	64.4 (125)	53.6 (104)	0.093
Marital status					<0.001^b^
Married	42.8 (253)	24.6 (50)	60.9 (117)	43.9 (86)	
Previously married	16.8 (99)	21.7 (44)	8.3 (16)	39 (19.9)	
Single (never married)	40.4 (239)	53.7 (109)	30.7 (59)	71 (36.2)	
Employment status					<0.001^b^
Employed	53.5 (315)	45.8 (92)	56.5 (109)	58.5 (114)	
Unemployed	27.5 (162)	40.3 (81)	17.6 (34)	24.1 (47)	
Homemaker/retired/student	19.0 (112)	13.9 (28)	25.9 (50)	17.4 (34)	
Family income					<0.001^b^
≤$15,999	44.1 (243)	56.7 (117)	41.4 (72)	29.8 (54)	
$16,000–$34,999	139 (25.2)	25.0 (49)	25.3 (44)	25.4 (46)	
$35,000–$74,999	20.7 (114)	12.2 (24)	23.0 (40)	27.6 (50)	
≥$75,00	10.0 (55)	3.1 (6)	10.3 (18)	17.1 (31)	
Education level					<0.001^b^
Less than high school	9.8 (58)	9.3 (19)	16.6 (32)	3.6 (7)	
High school/GED	53.6 (318)	63.4 (130)	48.7 (94)	48.2 (94)	
Trade school/Associate's degree	17.9 (106)	17.6 (36)	16.6 (32)	19.5 (38)	
Bachelor's degree and above	18.7 (111)	9.8 (20)	18.1 (35)	28.7 (56)	
Insurance status					<0.001^b^
Private insurance	42.3 (270)	39.1 (72)	45.6 (88)	56.7 (110)	
Medicare/Medicaid	29.3 (167)	42.9 (79)	24.4 (47)	21.1 (41)	
No insurance	23.5 (134)	17.9 (33)	30.1 (58)	22.2 (43)	
Current personal doctor					0.389
Yes	76.3 (447)	73.4 (146)	76.3 (148)	79.3 (148)	
No	23.7 (139)	26.6 (53)	23.7 (46)	20.7 (40)	
Annual visits					0.083
0–2	50.1 (265)	41.3 (66)	54.1 (98)	53.7 (101)	
3–5	32.0 (169)	38.1 (61)	30.9 (56)	27.7 (52)	
≥6	18.0 (95)	20.6 (33)	14.9 (27)	18.6 (35)	
Health care setting					<0.001^b^
Doctor's office	42.3 (200)	30.7 (50)	41.6 (64)	55.1 (86)	
Hospital/health system clinic	33.2 (157)	36.8 (60)	33.8 (52)	28.9 (45)	
Community clinic	18.6 (88)	21.5 (35)	21.4 (33)	12.8 (20)	
Urgent care/emergency	5.9 (28)	11.0 (18)	3.3 (5)	3.2 (5)	
Hard to get health services?					
Hard	37.1 (218)	45.0 (90)	40.3 (77)	26.0 (51)	0.104
Not very hard	52.8 (310)	49.0 (98)	46.6 (89)	62.8 (123)	
Have not needed HC in the last 2 years	10.1 (59)	6.0 (12)	13.1 (25)	11.2 (22)	
Not seek care because it was too expensive?					0.406
No	54.5 (318)	52.0 (104)	52.4 (318)	59.1 (114)	
Yes	37.8 (221)	39.5 (79)	38.2 (73)	35.8 (69)	
Not sure	7.7 (45)	8.5 (17)	9.4 (18)	5.2 (10)	
How quickly could you get an appointment					
<6 Days	77.0 (451)	77.5 (155)	76.0 (146)	77.3 (150)	0.318
>6 Days/never able/emergency	19.3 (113)	21.0 (42)	18.8 (36)	18.0 (35)	
Did not seek care in the last 2 years	3.8 (22)	1.5 (3)	5.2 (10)	4.6 (9)	
Did not follow advice/treatment plan because it cost too much?					0.058
Yes	27.9 (167)	25.5 (53)	30.4 (59)	28.1 (55)	
No	56.0 (104)	55.4 (111)	53.6 (104)	61.2 (120)	
Do not know	16.1 (96)	21.2 (44)	16.0 (31)	10.7 (21)	

Unless otherwise indicated, all values in the table include the percentage first followed by the absolute number in parenthesis.

^a^ *p*-Values measure any difference in three racial/ethnic groups.

^b^ Significant at *p*<0.001.

HC, health care.

Non-Hispanic blacks were more likely to have Medicare/Medicaid compared with Mexican Hispanics and non-Hispanic whites. Across all racial and ethnic groups ∼76% of our sample had a personal doctor. In comparison to the other two groups, non-Hispanic black respondents were also more likely to visit a physician >3 times annually. Non-Hispanic white and Mexican Hispanic respondents were more likely to have their usual source of health care to be in a physician's office compared with non-Hispanic blacks.

**FIG. 1.**
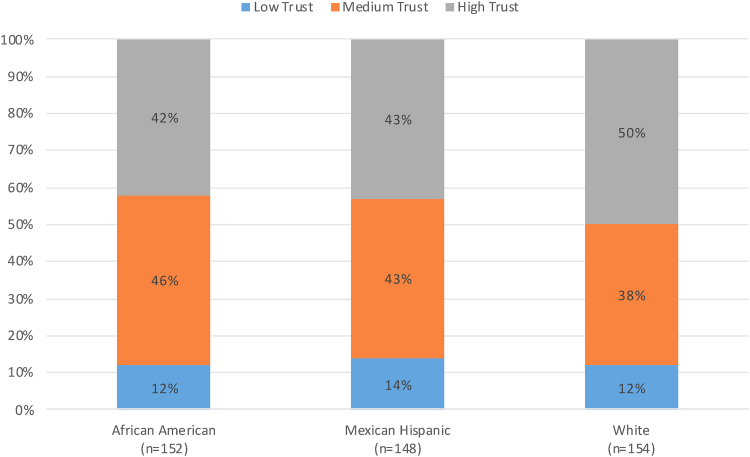
Percentage of people with low, medium, and high trust in each racial/ethnic group.

The unadjusted mean interpersonal trust (higher values equal lower interpersonal trust) across all racial/ethnic groups was 21.8 with a standard deviation (SD) of 7.5. Non-Hispanic blacks had a score of 22.1 (SD 7.0), Mexican Hispanics had a score of 22.5 (SD 7.4), and non-Hispanic whites had a score of 20.9 (SD 7.8). These differences were not statistically significant. Figure 1 shows the distribution of high, medium, and low interpersonal trust across the three racial/ethnic groups; 13%, 42%, and 45% had low, medium, and high trust, respectively. There was no difference in interpersonal trust categories across the three racial and ethnic groups (*p*=0.618).

Table 3 describes the results from the ordinal logistic regression. There was no significant relationship between race/ethnicity and the distribution of trust in any of the models. In Model 1, for every year that age increased, there was 0.98 decreased odds of lower interpersonal trust (odds ratio [OR]: 0.98; 95% confidence interval [CI] 0.97–1.00). In addition, reporting a family income between $35,000 and $74,999 was significantly associated with decreased odds of lower interpersonal trust compared with those with family incomes <$15,999 (OR: 0.48; 95% CI 0.27–0.85 and OR: 0.24; 95% CI 0.11–0.52, respectively).

**Table 3.** Odds of Low Interpersonal Trust in Three Logistic Regression Models Among Participants with a Personal Doctor

	Model 1(*n*=454),OR (95% CI), *p*-value	Model 2(*n*=454),OR (95% CI), *p*-value	Model 3 (*n*=454),OR (95% CI), *p*-value
Race/ethnicity			
Non-Hispanic White	Ref	Ref	Ref
Non-Hispanic Black	0.95 (0.59–1.52), 0.829	0.99 (0.61–1.60), 0.953	1.09 (0.66–1.79), 0.744
Mexican Hispanic	1.06 (0.67–1.68), 0.804	1.06 (0.66–1.69), 0.822	1.17 (0.72–1.91), 0.515
Age	0.98 (0.97–1.00), 0.032^*^	0.99 (0.97–1.00), 0.144	0.99 (0.98–1.01), 0.469
Female	0.96 (0.66–1.40), 0.822	1.04 (0.70–1.54), 0.848	1.07 (0.71–1.61), 0.738
Marital status			
Married	1.19 (0.76–1.85), 0.442	1.23 (0.78–1.93), 0.371	1.13 (0.70–1.81), 0.622
Previously married	0.81 (0.46–1.42), 0.4666	0.80 (0.45–1.41), 0.439	0.76 (0.42–1.37), 0.359
Single (never married)	Ref	Ref	Ref
Employment status			
Employed	Ref	Ref	Ref
Unemployed	1.18 (0.74–1.90), 0.485	1.26 (0.76–2.07), 0.366	1.22 (0.73–2.05), 0.453
Homemaker/retired/student	0.96 (0.59–1.57), 0.868	0.92 (0.55–1.54), 0.747	1.11 (0.65–1.90), 0.709
Family income (%)			
≤$15,999	Ref	Ref	Ref
$16,000–$34,999	0.83 (0.51–1.36), 0.466	0.77 (0.46–1.28), 0.310	0.75 (0.44–1.26), 0.276
$35,000–$74,999	0.48 (0.27–0.85), 0.011^*^	0.42 (0.23–0.78), 0.006^*^	0.54 (0.28–1.02), 0.059
≥$75,00	0.24 (0.11–0.52), <0.001^**^	0.21 (0.09–0.48)<0.001^**^	0.34 (0.14–0.79), 0.012^*^
Do not know/no response	0.92 (0.42–1.98), 0.824	0.89 (0.40–1.95), 0.763	0.87 (0.39–1.95), 0.734
Education level (%)			
Less than high school	0.91 (0.42–1.96), 0.809	0.78 (0.36–1.71), 0.542	0.78 (0.35–1.74), 0.545
High school/GED	0.89 (0.53–1.47), 0.640	0.80 (0.47–1.34), 0.399	0.84 (0.49–1.44), 0.515
Trade school/Associate's degree	0.83 (0.45–1.54), 0.562	0.78 (0.42–1.45), 0.431	0.81 (0.43–1.53), 0.507
Bachelor's degree and above	Ref	Ref	Ref
Insurance status			
Private insurance	N/A	Ref	Ref
Medicare/Medicaid	N/A	0.83 (0.51–1.37), 0.473	0.75 (0.45–1.26), 0.281
No insurance	N/A	0.96 (0.54–1.70), 0.880	0.58 (0.30–1.09), 0.090
No. of annual visits			
0–2	N/A	1.84 (1.11–3.04), 0.018^*^	1.89 (1.12–3.19), 0.017^*^
3–5	N/A	1.07 (0.65–1.79), 0.784	0.94 (0.55–1.59), 0.812
≥6	N/A	Ref	Ref
Health care setting			
Doctor's office	N/A	Ref	Ref
Hospital/health system clinic	N/A	0.94 (0.61–1.45), 0.779	0.80 (0.51–1.26), 0.345
Community clinic	N/A	1.44 (0.85–2.42), 0.172	1.08 (0.62–1.87), 0.790
Urgent care/emergency	N/A	2.40 (1.04–5.50), 0.039^*^	2.06 (0.85–4.94), 0.107
Hard to get health services			
Hard	N/A	N/A	1.77 (1.05–2.97), 0.032^*^
Not very hard	N/A	N/A	Ref
Have not needed HC in last 2 years	N/A	N/A	1.04 (0.52–2.08), 0.909
Did not seek care because too expensive			
No	N/A	N/A	Ref
Yes	N/A	N/A	1.21 (0.72–2.01), 0.476
Not sure	N/A	N/A	1.61 (0.73–3.56), 0.243
Time to getting appointment			
<6 Days	N/A	N/A	Ref
>6 Days/never able/emergency	N/A	N/A	2.73 (1.55–4.82), 0.001^*^
Did not seek care	N/A	N/A	2.32 (0.94–5.74), 0.068
Did not follow advice because cost too much			
Yes	N/A	N/A	2.48 (1.51–4.07), <0.001^**^
No	N/A	N/A	Ref
Do not know	N/A	N/A	2.26 (0.92–5.56), 0.076

^*^ Significant at *p*<0.05.

^**^ Significant at *p*<0.001.

CI, confidence interval; OR, odds ratio.

In Model 2 the relationship between income and trust remained significant. In addition, having 0–2 annual doctor visits compared with >6 visits was associated with increased odds of lower interpersonal trust (OR 1.84; 95% CI 1.11–3.04). Obtaining health care at an urgent care clinic/emergency room compared with a doctor's office was significantly associated with increased odds of lower interpersonal trust (OR 2.40; 95% CI 1.04–5.50).

In the fully adjusted Model 3, having an income greater than $75,000 (vs. less than $15,999) was still significantly associated with decreased odds of lower interpersonal trust, and having 0–2 annual doctor visits (vs. >6 visits) was associated with increased odds of lower interpersonal trust. In addition, respondents that reported having a “hard time” getting health care services in the past 2 years (vs. those that answered “not very hard”: OR 1.77; 95% CI 1.05–2.97), who reported waiting more than 6 days, never getting an appointment (vs. those who did not; OR 2.73; CI 1.55–4.82) and who answered “yes” to “Did you not follow advice or treatment plan because it cost too much?” (vs. “no”; OR 2.48; 95% CI 1.51–4.07), were significantly associated with increased odds of low trust. The sensitivity analysis did not change these results (Supplementary Table S1).

Table 4 describes the odds of lower interpersonal trust in the three different racial/ethnic groups. For non-Hispanic blacks, having Medicare/Medicaid or no insurance was associated with decreased odds of lower interpersonal trust compared with private insurance (OR 0.36; 95% CI 0.13–0.96 and OR 0.25; 95% CI 0.07–0.93, respectively). Additionally, non-Hispanic blacks who said “yes” they did not follow advice because it cost too much had 4.62 increased odds (CI 1.85–11.53) of lower interpersonal trust compared with those who responded “no.”

**Table 4.** Odds of Low Interpersonal Trust in Three Different Racial/Ethnic Groups

	Non-Hispanic Black (*n*=152), OR (95% CI), *p*-value	Mexican Hispanic (*n*=148), OR (95% CI), *p*-value	Non-Hispanic White (*n*=154), OR (95% CI), *p*-value
Age	1.02 (0.98–1.06), 0.276	1.01 (0.98–1.04), 0.405	0.97 (0.94–1.00), 0.071
Female	1.39 (0.60–3.24), 0.446	0.96 (0.44–2.08), 0.908	1.38 (0.63–3.01), 0.420
Marital status			
Married	1.37 (0.51–3.70), 0.530	1.15 (0.48–2.75), 0.758	0.89 (0.37–2.14), 0.79
Previously married	0.56 (0.20–1.54), 0.258	0.73 (0.15–3.56), 0.697	0.44 (0.14–1.41), 0.167
Single (never married)	Ref	Ref	Ref
Employment status			
Employed	Ref	Ref	Ref
Unemployed	2.20 (0.80–6.05), 0.127	1.32 (0.46–3.79), 0.604	0.87 (0.27–2.70), 0.805
Homemaker/retired/student	1.35 (0.43–4.27), 0.612	1.50 (0.58–3.85), 0.403	0.64 (0.19–2.13), 0.468
Family income (%)			
≤$15,999	Ref	Ref	Ref
$16,000–$34,999	0.40 (0.16–0.98), 0.045^*^	1.23 (0.43–3.55), 0.696	0.97 (0.30–3.12), 0.953
$35,000–$74,999	0.29 (0.07–1.11), 0.071	0.74 (0.22–2.55), 0.638	0.79 (0.22–2.90), 0.723
≥$75,00	0.18 (0.02–1.52), 0.114	0.49 (0.09–2.66), 0.409	0.36 (0.80–1.62), 0.184
Do not know/no response	0.44 (0.063–3.10), 0.411	1.89 (0.45–7.90), 0.385	0.48 (0.96–2.37), 0.367
Education level (%)			
Less than high school	0.54 (0.09–3.12), 0.492	0.39 (0.09–1.60), 0.190	0.623 (0.84–4.60), 0.643
High school/GED	0.67 (0.21–2.12), 0.496	0.55 (0.17–1.75), 0.310	0.74 (0.28–1.99), 0.550
Trade school/Associate's degree	0.84 (0.22–3.14), 0.791	0.60 (0.18–1.97), 0.401	0.53 (0.16–1.82), 0.314
Bachelor's degree and above	Ref	Ref	Ref
Insurance status			
Private insurance	Ref	Ref	Ref
Medicare/Medicaid	0.36 (0.133–0.96), 0.041^*^	1.05 (0.3802.87), 0.929	1.40 (0.50–3.91), 0.518
No insurance	0.25 (0.07–0.93), 0.038^*^	0.73 (0.21–2.55), 0.624	0.37 (0.10–1.36), 0.134
No. of annual visits			
0–2	1.59 (0.59–4.31), 0.803	4.00 (1.41–11.36), 0.009^*^	1.59 (0.53–4.72), 0.83
3–5	0.71 (0.29–1.78), 0.470	1.11 (0.36–3.36), 0.858	1.13 (0.38–3.35), 0.21
≥ 6	Ref	Ref	Ref
Health care setting			
Doctor's office	Ref	Ref	Ref
Hospital/health system clinic	0.89 (0.36–2.22), 0.803	0.72 (0.28–1.85), 0.496	0.54 (0.21–1.37), 0.195
Community clinic	1.41 (0.50–3.98), 0.520	0.54 (0.20–1.47), 0.227	5.44 (1.5–19.18), 0.008^*^
Urgent care/emergency	2.04 (0.54–7.69), 1.05	2.03 (0.20–20.75), 0.552	20.07 (1.91–210.56), 0.012^*^
Hard to get health services			
Hard	0.85 (0.33–2.18), 0.737	3.17 (1.16–8.65), 0.025^*^	2.92 (0.85–10.08), 1.70
Not very hard	Ref	Ref	Ref
Have not needed HC in last 2 years	0.99 (0.19–5.21), 0.994	0.82 (0.18–3.88), 0.807	0.67 (0.14–3.17), 0.612
Did not seek care because too expensive			
No	Ref	Ref	Ref
Yes	1.33 (0.52–3.42), 0.548	1.07 (0.38–3.02), 0.902	1.83 (1.05–11.8), 0.041^*^
Not sure	4.94 (0.911–26.79), 0.064	0.82 (0.18–3.88), 0.807	0.59 (0.10–3.38), 0.555
Time to getting appointment			
<6 Days	Ref	Ref	Ref
>6 Days/never able/emergency	2.49 (0.84–7.40), 0.101	4.98 (1.47–16.83), 0.010)^*^	3.53 (1.05–11.82), 2.04
Did not seek care	0.99 (0.19–5.21), 0.994	20.28 (2.65–155.18), 0.004^*^	2.68 (0.40–17.80), 0.308
Did not follow advice because cost too much			
Yes	4.62 (1.85–11.53), 0.001^*^	1.05 (0.39–2.83), 0.930	2.09 (0.76–5.79), 0.153
No	Ref	Ref	Ref
Do not know	3.12 (0.78–12.47), 0.107	2.31 (0.32–16.72), 0.407	20.2 (1.47–278.72), 0.025^*^

^*^ Significant at *p*<0.05.

^**^ Significant at *p*<0.001.

For Mexican Hispanics, having 0–2 annual visits (vs. >6; OR 4.00; CI 1.41–11.36), reporting that it was “hard” to get health services (vs. not hard; OR 3.17; CI 1.16–8.65) and having greater than six days wait for an appointment (vs. <6, OR 4.98; CI 1.47–16.83), all had increased odds of lower interpersonal trust.

For non-Hispanic whites, going to a community clinic (vs. doctor's office OR 5.44; CI 1.5–19.18) and waiting greater than 6 days for an appointment (vs. <6 days, OR 3.53; CI 1.05–11.82), all had increased odds of lower interpersonal trust.

## Discussion

Unlike previous studies, we did not find significant differences in interpersonal trust across racial/ethnic groups. Instead, we found that reporting fewer annual physician visits (0–2), having a difficult time accessing health services in a timely manner, and not following a physician's advice due to cost were significantly associated with lower interpersonal trust. Having a higher income significantly decreased the odds of low interpersonal trust.

We hypothesized that we would find differences in interpersonal trust between racial and ethnic groups because this relationship existed in previous studies.^6,11,20,23^ We might not have seen a difference in interpersonal trust in this study because of the unique way in which we recruited a community sample or because racial/ethnic differences in physician trust vary geographically. Previous studies have shown that the size of the difference in distrust among racial/ethnic groups varies across cities and sociodemographic groups.^16^ It is also possible that the reason why we did not see differences in trust across racial/ethnic groups is because other modifiable factors may be more important than one's self-identified race/ethnicity in trusting one's physician.

Although we did not see differences in interpersonal trust across racial/ethnic groups, the subgroup analysis shows that the access-to-care variables and health care utilization variables that influence interpersonal trust are unique to each racial/ethnic group. For non-Hispanic blacks, the cost of care was the most associated with lower trust; for Mexican Hispanics, access to care and utilization were the most strongly associated factors; for whites, office setting and wait times were the factors most associated with lower trust. These results suggest that the factors influencing interpersonal trust are unique to each racial/ethnic group and that efforts to enhance trust may need to be tailored for different groups.

We found that measures of health care utilization and access to interpersonal trust are associated with trust, confirming our hypothesis that greater difficulty accessing health care would be associated with lower physician trust. This is in keeping with previous studies that found that receiving care in a physician's office, number of quality interactions with a personal physician, and lack of continuity with a personal physician more than sociodemographic factors, including insurance status, were significantly associated with trust levels.^10,11,19,20,23^

This work is unique because we included a large Mexican Hispanic population. Most studies of interpersonal trust have only included non-Hispanic blacks and non-Hispanic white participants. Our study is among the first to compare interpersonal trust scores using a validated scale between Mexican Hispanic respondents and non-Hispanic blacks and non-Hispanic whites. Armstrong et al. used 4 items from the Community-tracking survey to measure physician distrust and found that Hispanics had higher physician distrust than whites, but that this relationship varied across geographic region.^16^ So these results may not be generalizable to other Hispanic identifying populations.

Previous work highlights that the interpersonal trust and health outcome relationship is likely more complex than a simple cause and effect feedback loop.^24^ In fact, a major topic discussed at the American Board of Internal Medicine Foundation annual retreat in 2018 focusing on trust in physicians, was that there is no single approach that will ensure, maintain, or rebulild trust.^25^ It could be that increased access leads to increased trust; however, it could also be that by improving interpersonal trust perceptions about health care access could change and, as our findings suggest, different factors influence trust in different communities and populations.

There are several factors that may limit the ability to generalize the study findings. We recruited a convenience sample of Chicago residents, so our results may not be generalizable to the United States. Additionally, our study sample population had relatively low educational attainment and income levels. A more diversified population sample could have revealed higher trust disparities between the three racial/ethnic groups.

The study was a cross-sectional study and therefore causal relationships cannot be established. We could not and did not measure all factors that may influence trust in one's physician, including the physician's trust in the patient and cultural competence, and immigration status (due to the sensitivity of this question) and we lost some information by using categories, such as with income, instead of using a continuous variable. We also did not explore the impact of race within the Mexican Hispanic group as very few of our participants chose a race other than white.

The study also had several strengths. As mentioned previously, we included participants identifying as Mexican Hispanic in our sample and did not treat “Hispanic” as a monolithic group. Second, we included measures of experience accessing medical care rather than just insurance status and number of annual visits. Third, we surveyed respondents outside of a health care setting, which may have allowed respondents to answer more honestly.

### Health equity implications

Our study contributes to the growing body of literature that examines the underlying determinants of interpersonal trust. Our results suggest that access to health care and the types of interactions a person has within the health care setting may have the strongest impact on interpersonal trust with one's physician. Additionally, we found that the factors that seem to have the greatest relationship with interpersonal trust vary by racial/ethnic group. Therefore, to enhance interpersonal trust in health care, it is important to take multifaceted and tailored approaches so that interpersonal trust improves among all racial/ethnic groups.

To understand more about how to promote equity in trust, we endorse The American Board of Internal Medicine Foundation's recommendations for increasing trust among patients and the organizations and teams that care for them.^25^ Particularly we support incorporating measurements of trust into the standard evaluation of patient care experiences so that organizations have a real-time understanding of patients' trust in their providers and how it might differ among groups that experience discrimination and health care disparities. We also support education of physicians that emphasizes communication and relationship skills. Future research should explore what strategies designed to improve interpersonal trust work best in all different types of underrepresented groups, including racial and ethnic minorities.

## Supplementary Material

Supplemental data

## Abbreviations Used

CI: confidence interval
OR: odds ratio
SD: standard deviation

## References

[1] Thom DH, Hall MA, Pawlson LG. Measuring patients' trust in physicians when assessing quality of care. Health Aff Proj Hope. 2004;23:124–132 10.1377/hlthaff.23.4.124 15318572

[2] Calnan M, Rowe R. Researching trust relations in health care: conceptual and methodological challenges—introduction. J Health Organ Manag. 2006;20:349–358 17087399 10.1108/14777260610701759

[3] Hall MA, Camacho F, Lawlor JS, et al. Measuring trust in medical researchers. Med Care. 2006;44:1048–1053 17063137 10.1097/01.mlr.0000228023.37087.cb

[4] Hall MA, Dugan E, Zheng B, et al. Trust in physicians and medical institutions: what is it, can it be measured, and does it matter? Milbank Q. 2001;79:613–639, v. 11789119 10.1111/1468-0009.00223 PMC2751209

[5] Blackstock OJ, Addison DN, Brennan JS, et al. Trust in primary care providers and antiretroviral adherence in an urban HIV clinic. J Health Care Poor Underserved. 2012;23:88–98 10.1353/hpu.2012.0006 22643464

[6] Kaiser K, Rauscher GH, Jacobs EA, et al. The import of trust in regular providers to trust in cancer physicians among white, African American, and Hispanic breast cancer patients. J Gen Intern Med. 2011;26:51–57 20811783 10.1007/s11606-010-1489-4 PMC3024096

[7] Saha S, Jacobs EA, Moore RD, et al. Trust in physicians and racial disparities in HIV care. AIDS Patient Care STDs. 2010;24:415–420 20578909 10.1089/apc.2009.0288 PMC3472674

[8] Trachtenberg F, Dugan E, Hall MA. How patients' trust relates to their involvement in medical care. J Fam Pract. 2005;54:344–352 15833226

[9] Birkhäuer J, Gaab J, Kossowsky J, et al. Trust in the health care professional and health outcome: a meta-analysis. PLoS One. 2017;12:e0170988 28170443 10.1371/journal.pone.0170988 PMC5295692

[10] Boulware LE, Cooper LA, Ratner LE, et al. Race and trust in the health care system. Public Health Rep Wash DC 1974. 2003;118:358–365 10.1016/S0033-3549(04)50262-5 PMC1497554 12815085

[11] Doescher MP, Saver BG, Franks P, et al. Racial and ethnic disparities in perceptions of physician style and trust. Arch Fam Med. 2000;9:1156–1163 11115223 10.1001/archfami.9.10.1156

[12] Keating F, Robertson D. Fear, black people and mental illness: a vicious circle? Health Soc Care Community. 2004;12:439–447 15373823 10.1111/j.1365-2524.2004.00506.x

[13] LaVeist TA, Nickerson KJ, Bowie JV. Attitudes about racism, medical mistrust, and satisfaction with care among African American and white cardiac patients. Med Care Res Rev MCRR. 2000;57 Suppl 1:146–161 11092161 10.1177/1077558700057001S07

[14] Gordon HS, Street RL, Sharf BF, et al. Racial differences in trust and lung cancer patients' perceptions of physician communication. J Clin Oncol Off J Am Soc Clin Oncol. 2006;24:904–909 10.1200/JCO.2005.03.1955 16484700

[15] Stepanikova I, Mollborn S, Cook KS, et al. Patients' race, ethnicity, language, and trust in a physician. J Health Soc Behav. 2006;47:390–405 17240927 10.1177/002214650604700406

[16] Armstrong K, Ravenell KL, McMurphy S, et al. Racial/ethnic differences in physician distrust in the United States. Am J Public Health. 2007;97:1283–1289 17538069 10.2105/AJPH.2005.080762 PMC1913079

[17] Kayaniyil S, Gravely-Witte S, Stewart DE, et al. Degree and correlates of patient trust in their cardiologist. J Eval Clin Pract. 2009;15:634–640 19522723 10.1111/j.1365-2753.2008.01064.x PMC2972247

[18] White RO, Osborn CY, Gebretsadik T, et al. Health literacy, physician trust, and diabetes-related self-care activities in hispanics with limited resources. J Health Care Poor Underserved. 2013;24:1756–1768 24185168 10.1353/hpu.2013.0177 PMC3916094

[19] Carpenter WR, Godley PA, Clark JA, et al. Racial differences in trust and regular source of patient care and the implications for prostate cancer screening use. Cancer. 2009;115:5048–5059 19637357 10.1002/cncr.24539 PMC2779840

[20] Halbert CH, Armstrong K, Gandy OH, et al. Racial differences in trust in health care providers. Arch Intern Med. 2006;166:896–901 16636216 10.1001/archinte.166.8.896

[21] Hall MA, Zheng B, Dugan E, et al. Measuring patients' trust in their primary care providers. Med Care Res Rev MCRR. 2002;59:293–318 12205830 10.1177/1077558702059003004

[22] Little RJA, Rubin DA. Statistical Analysis with Missing Data, 2nd ed. Hoboken, New Jersey: John Wiley & Sons, Ltd, 2002

[23] Armstrong K, Rose A, Peters N, et al. Distrust of the health care system and self-reported health in the United States. J Gen Intern Med. 2006;21:292–297 16686803 10.1111/j.1525-1497.2006.00396.x PMC1484714

[24] Giordano GN, Lindström M. Trust and health: testing the reverse causality hypothesis. J Epidemiol Community Health. 2016;70:10–16 26546287 10.1136/jech-2015-205822 PMC4717376

[25] Lee TH, McGlynn EA, Safran DG. A Framework for increasing trust between patients and the organizations that care for them. JAMA. 2019;321:539–540 30676628 10.1001/jama.2018.19186

