# Supplementary material for: What Matters When It Comes to Trust in One's Physician: Race/Ethnicity, Sociodemographic Factors, and/or Access to and Experiences with Health Care?
Source: Health Equity. 2020 Jun 29;4(1):280–9. doi: 10.1089/heq.2019.0101 (PMC8175262; doi:10.1089/heq.2019.0101)
Supplement: Supplemental data [file Suppl_TableS1.pdf]

Supplementary Data

Supplementary Table S1. Odds of Low Interpersonal Trust in Three Logistic Regression Models Among Participants with a Personal Doctor with Expanded Categories for Number of Annual Visits and Time to Getting an Appointment

	Model 1 ($n=381$), OR (95% CI), p-value	Model 2 ($n=354$), OR (95% CI), p-value	Model 3 ($n=327$), OR (95% CI), p-value
Race/ethnicity			
Non-Hispanic White	Ref	Ref	Ref
Non-Hispanic Black	0.88 (0.52–1.50), 0.648	0.74 (0.42–1.30), 0.293	0.87 (0.45–1.65), 0.660
Mexican Hispanic	1.03 (0.63–1.67), 0.903	0.98 (0.59–1.62), 0.932	1.04 (0.59–1.86), 0.888
Age	0.99 (0.97–1.00), 0.077	0.98 (0.97–1.00), 0.053	0.99 (0.97–1.00), 0.137
Female	0.99 (0.66–1.49), 0.970	1.06 (0.68–1.65), 0.795	1.24 (0.76–2.02), 0391
Marital status			
Married	1.24 (0.76–2.03), 0.442	1.32 (0.79–2.21), 0.294	1.41 (0.79–2.52), 0.241
Previously married	0.84 (0.44–1.58), 0.579	0.77 (0.38–1.41), 0.439	1.17 (0.54–2.57), 0.690
Single (never married)	Ref	Ref	Ref
Employment status			
Employed	Ref	Ref	Ref
Unemployed	1.12 (0.67–1.87), 0.676	1.28 (0.72–2.25), 0.402	1.23 (0.64–2.39), 0.532
Homemaker/retired/student	1.06 (0.62–1.83), 0.829	0.91 (0.50–1.65), 0.752	1.25 (0.64–2.46), 0.513
Family income (%)			
≤ $15,999	Ref	Ref	Ref
$16,000–$34,999	0.89 (0.52–1.52), 0.657	0.92 (0.52–1.62), 0.763	0.85 (0.44–1.65), 0.626
$35,000–$74,999	0.45 (0.24–0.85), 0.013*	0.47 (0.23–0.94), 0.034*	0.46 (0.21–1.03), 0.058
≥ $75,00	0.25 (0.11–0.56), 0.001*	0.24 (0.10–0.58) 0.001*	0.34 (0.13–0.92), 0.034*
Do not know/no response	0.85 (0.36–2.01), 0.710	0.96 (0.38–1.53), 0.456	0.84 (0.31–2.29), 0.730
Education level (%)			
Less than high school	0.81 (0.35–1.85), 0.611	0.47 (0.18–1.21), 0.117	0.63 (0.21–1.95), 0.426
High school/GED	0.91 (0.53–1.57), 0.726	0.74 (0.42–1.33), 0.315	0.64 (0.34–1.20), 0.162
Trade school/Associate's degree	0.75 (0.39–1.47), 0.406	0.77 (0.39–1.53), 0.456	0.63 (0.30–1.33), 0.229
Bachelor's degree and above	Ref	Ref	Ref
Insurance status			
Private insurance	N/A	Ref	Ref
Medicare/Medicaid	N/A	0.98 (0.56–1.73), 0.951	0.67 (0.35–1.30), 0.238
No insurance	N/A	1.07 (0.56–2.03), 0.846	0.51 (0.23–1.13), 0.098
No. of annual visits		0.96 (0.92–1.01), 0.156	0.98 (0.93–1.04); 0.515
Health care setting			
Doctor's office	N/A	Ref	Ref
Hospital/health system clinic	N/A	0.90 (0.55–1.47), 0.671	0.65 (0.38–1.14), 0.134
Community clinic	N/A	1.61 (0.88–2.95), 0.121	1.11 (0.55–2.25), 0.771
Urgent care/emergency	N/A	2.67 (0.96–7.39), 0.059	3.59 (1.17–11.06), 0.026*
Hard to get health services			
Hard	N/A	N/A	2.13 (1.10–4.12), 0.024*
Not very hard	N/A	N/A	Ref
Have not needed HC in last 2 years	N/A	N/A	0.37 (0.10–1.36), 0.135
Did not seek care because too expensive			
No	N/A	N/A	Ref
Yes	N/A	N/A	1.15 (0.61–2.19), 0.661
Not sure	N/A	N/A	0.88 (0.30–2.62), 0.819
Time to getting appointment			
Same day	N/A	N/A	Ref
Next day	N/A	N/A	1.58 (0.82–3.03); 1.37
2–3 Days	N/A	N/A	1.22 (0.62–2.39); 0.573
4–5 Days	N/A	N/A	3.72 (1.25–11.03); 0.018*
6.7 Days	N/A	N/A	9.28 (3.05–28.22); < 0.001**
> 1 Week	N/A	N/A	5.23 (1.79–15.25); 0.002*
Never got appointment	N/A	N/A	3.8 (0.34–41.40); 0.278
Went to ER/urgent care because could not get appointment	N/A	N/A	3.36 (0.69–16.26); 0.133
Did not seek care	N/A	N/A	1.96 (0.47–8.06); 0.353
Did not follow advice because cost too much			
Yes	N/A	N/A	2.08 (1.11–3.92), 0.023*
No	N/A	N/A	Ref
Do not know	N/A	N/A	2.12 (0.58–7.71); 0.254

*Significant at $p < 0.05$.
**Significant at $p < 0.001$.
ER, emergency room; HC, health care.
